# Construction and validation of a fall risk prediction model in elderly maintenance hemodialysis patients: a multicenter prospective cohort study

**DOI:** 10.1080/0886022X.2025.2455524

**Published:** 2025-02-17

**Authors:** Lin Li, Wenbin Xu, Yiqian Fang, Qian Jiang, Yanfei Zhou, Yan Chen, Qian Yang

**Affiliations:** a School of Nursing, Chengdu Medical College, Chengdu, China; b Department of Neonatal Intensive Care Unit, Sichuan Provincial People’s Hospital, Chengdu, China

**Keywords:** Fall, hemodialysis, risk prediction model, risk factors

## Abstract

**Purpose:**

To analyze the risk factors for falls in elderly maintenance hemodialysis patients, construct a nomogram prediction model and validate the application.

**Background:**

Elderly maintenance hemodialysis patients face a high risk of falls, and there are fewer and less effective fall-specific assessment tools.

**Method:**

A total of 871 elderly hemodialysis patients from 9 hospitals in Chengdu City from October 2023 to December 2024 were selected as the study objects. Baseline characteristics and fall outcomes of patients in the fall group and non-fall group were recorded and compared through 6-month follow-up. Multivariable logistic regression analysis was employed to identify independent risk factors, and construct the nomogram prediction model and complete the internal verification of the model. 218 elderly maintenance hemodialysis patients from three other hospitals in Chengdu City were selected for a 6-month follow-up of falls from January to February 2024 to complete the external validation of the model.

**Result:**

The incidence of falls in elderly maintenance hemodialysis patients was 31.96%, and logistic regression analysis showed that age, sex, visual impairment, intradialytic hypotension, cognitive impairment and depression were independent risk factors for falls. Both internal and external validation of the model demonstrated area under the curve greater than 0.80. Furthermore, calibration plots, the Hosmer-Lemeshow test, and clinical decision curves all demonstrated that the model had good calibration and clinical utility.

**Conclusion:**

The nomogram constructed based on the above risk factors can provide scientific basis and practical tools for early clinical identification of high-risk groups of falls.

## 1. Introduction

Maintenance Hemodialysis (MHD), as the most commonly used renal replacement therapy for patients with end-stage renal disease, can effectively prolong the survival of patients, but with the accumulation of dialysis time, elderly MHD patients are often accompanied by complications such as weakness, malnutrition, decreased muscle mass, and cognitive decline, which are intertwined with each other and greatly increase the risk of falls in patients [1,2]. As one of the most common adverse events in the elderly, falls may not only lead to disability, but even become an important cause of death [3]. It has been reported that the incidence of falls in elderly MHD patients is 12% to 49%, which is twice that of healthy people in the community, and the risk of death within one year after falls is 60% higher than that of those without falls [4,5], posing a serious threat to the quality of life of patients.

Because of the serious consequences of falls, it is particularly important to adopt effective fall risk assessment tools for early screening and intervention in elderly MHD patients [6]. Although a variety of fall risk assessment tools have been applied to different populations and scenarios, the specific assessment tools for hemodialysis patients are still insufficient. In addition to the general risk of falls, hemodialysis patients also face risk factors specific to the dialysis process, such as intradialytic hypotension. Therefore, the direct application of the fall assessment scale in non-hemodialysis elderly patients to elderly MHD patients may lack pertinence and accuracy. Based on this, through a multicenter, prospective study design, this study deeply analyzed the potential influencing factors of falls in elderly MHD patients, built a visual nomogram risk prediction model based on these factors, and converted it into an easy-to-use web calculator, to provide scientific reference for early identification and accurate prevention of falls in elderly MHD patients.

## 2. Method

### 2.1. Study design and participants

In this prospective cohort study, elderly MHD patients admitted to 9 blood purification centers in Chengdu from October 2023 to December 2024 were selected as the modeling group, and elderly MHD patients admitted to 3 other blood purification centers in Chengdu from January 2024 to February 2024 were selected as the validation group.

Inclusion criteria: (1) age ≥ 60 years; (2) patients diagnosed with end-stage renal disease (ESRD) in accordance with the guidelines established by the Kidney Disease Outcome Quality Initiative (KDOQI) [7] and the diagnostic criteria outlined in the “Guidelines for Screening, Diagnosis, and Prevention of Chronic Kidney Disease” [8], and who had received regular hemodialysis treatment for at least 3 months. Exclusion criteria: (1) serious comorbidities such as severe infections, organ failure, or malignant tumors; (2) neurological disorders; (3) language or communication barriers that hinder understanding or response to questions.

Finally, this study successfully recruited 1,283 elderly MHD patients from 12 blood purification centers in Chengdu as research objects, excluded 142 patients who did not meet the inclusion criteria, and finally included a total sample size of 1141 cases.

### 2.2. Ethics statement

Ethical approval for this study was obtained from the Ethics Committee of Chengdu Medical College (Ethics approval number: CMCEC 2023. NO.122), and the study strictly adhered to the principles outlined in the Declaration of Helsinki. All participants provided informed consent.

### 2.3. Study tools

#### 2.3.1. Demographic characteristics

By reviewing the literature, consulting with experts in the relevant fields, designing their questionnaires. This questionnaire encompasses general patient information, including age, sex, education, marital status, BMI, dialysis age, drinking history, smoking history and exercise. Furthermore, it includes treatment factors such as visual impairment, hearing impairment, complications, sedative hypnotic drugs, intradialytic hypotension, among them, intradialytic hypotension refers to the decrease of systolic blood pressure ≥20mmHg, or the decrease of mean arterial pressure ≥10mmHg during dialysis, accompanied by hypotension-related symptoms such as headache, general fatigue, nausea and restlessness during dialysis. Laboratory examination indicators including total protein, hemoglobin (Hb), albumin (Alb), hypersensitive c-reactive protein, triglycerides (TG), total cholesterol (TC), urea, glomerular filtration rate (GFR), serum creatinine, potassium (K), calcium (Ca), sodium (Na), and phosphorus (P).

#### 2.3.2. Frailty assessment

The Frailty Phenotype Scale was utilized to evaluate the frailty status of patients. It was developed by Fried in 2001 and includes five indicators: weight loss, slowed walking speed, low grip strength, low physical activity, and fatigue. The total score ranges from 0 to 5, with scores of 3 to 5 indicating frailty, scores of 1 to 2 indicating pre-frailty, and a score of 0 indicating no frailty. This scale has been widely applied in assessing frailty among patients undergoing hemodialysis. The Cronbach’s alpha coefficient for this scale is 0.93 [9,10].

#### 2.3.3. Cognitive function assessment

The Montreal Cognitive Assessment (MoCA) Scale [11] is utilized to evaluate eight cognitive domains, including abstract thinking, attention and concentration, executive function, language, memory, visuospatial skills, calculation, and orientation. The total score ranges from 0 to 30, with higher scores indicating better cognitive function; a score of 26 or above is considered indicative of normal cognitive function. For participants with less than 12 years of education, 1 point is added to the total score to correct for educational bias. The Cronbach’s alpha coefficient for this scale is 0.81 [12].

#### 2.3.4. Depressive symptom

The 15-Item Geriatric Depression Scale (GDS-15) is employed to assess the depressive status of patients. It was developed by Sheikh and Yesavage to specifically address the characteristics of the elderly, primarily evaluating symptoms such as low mood, reduced activity, irritability, withdrawal, and negative evaluations concerning past, present, and future experiences. The scale consists of 15 items, with a total score ranging from 0 to 15. Patients are instructed to respond with “yes" or “no” to each item, scoring 1 point for “yes” and 0 points for “no”. A total score of ≥8 indicates the presence of depressive symptoms, with higher scores reflecting more severe depressive symptoms. The Cronbach’s alpha coefficient for this scale is 0.82 [13,14].

#### 2.3.5. Nutritional status

The Malnutrition Inflammation Score (MIS) Scale, developed by Kalantar-Zadeh [15], is specifically designed to assess the nutritional status of patients with chronic kidney disease undergoing dialysis. The scale consists of four components: medical history, physical examination, body mass index (BMI), and laboratory tests, comprising a total of 10 items. Each item is scored from 0 to 3, resulting in a total score ranging from 0 to 30, with higher scores indicating a greater risk of malnutrition [16].

#### 2.3.6. Sleep quality

The Pittsburgh Sleep Quality Index (PSQI) Scale is utilized to assess patients’ sleep quality. Developed by Buysse [17] in 1989, it encompasses seven components: subjective sleep quality, sleep latency, sleep duration, sleep efficiency, sleep disturbances, use of sleeping medication, and daytime dysfunction, totaling 18 items. Scores range from 0 to 21, with a score of 7 or higher indicating the presence of sleep problems. The Cronbach’s alpha coefficient for this scale is 0.7962 [18].

#### 2.3.7. Activities of daily living

The Activities of Daily Living Scale, developed by Mahoney [19], is employed to assess patients’ ability to perform daily activities, including bowel control, bladder control, grooming, toileting, eating, bathing, dressing, transfers from bed to chair, ambulation on level surfaces, and climbing stairs, comprising a total of 10 items. The scoring ranges from 0 to 100, with a score of 40 indicating severe dependence, requiring complete care from others; scores of 41 to 60 indicating moderate dependence, necessitating most care from others; scores of 61 to 99 indicating mild dependence, requiring some assistance; and a score of 100 indicating independence, with the individual fully capable of self-care. The Cronbach’s alpha coefficient for this scale is 0.88 [20].

#### 2.3.8. Social support

The Social Support Rating Scale (SSRS) was compiled by Chinese scholar Xiao Shui yuan in 1994, assesses social support across three dimensions: objective support, subjective support, and utilization of social support. The scale comprises a total of 10 items, with scores ranging from 12 to 66. Higher scores indicate a greater level of social support, with a score of 22 denoting low support, 23 to 44 indicating moderate support, and 45 or above representing high support. It has been proven to have good reliability and validity in hemodialysis patients, with Cronbach’s alpha coefficients of 0.825 to 0.896 [21].

### 2.4. Assessment of falls

According to the currently accepted definition of fall by the European Fall Prevention Collaboration Network, fall is defined as falling unintentionally on the ground, floor or other low level, excluding intentional changes in one’s own position [3]. Taking the questionnaire as the starting point, the researchers followed up the patients’ falls by telephone. They contacted the patients once every 2 months for a total of 3 follow-up visits, including the time, place and reason of falls. According to the follow-up results, the patients were divided into fall group and non-fall group.

### 2.5. Data collection methods

To carry out this study, we set up a professional research team consisting of three nursing graduate students and five nurses with intermediate or higher titles. (1) The demographic characteristics, frailness and sleep status of the patients were collected through questionnaire survey. The research team explained the purpose, content and filling method of the questionnaire to the study subjects after unified training. The researchers should complete the questionnaire independently. After the questionnaire is returned, double check for omissions and filling errors. (2) Extract disease-related information and laboratory examination indicators from the hospital health information system (HIS), in which the laboratory examination indicators take the patient’s latest examination results, and after extracting the data, two members of the team will double-check to ensure the accuracy of the data.

### 2.6. Statistical analysis

IBM SPSS 26.0 software and R4.1.2 were used for statistical analysis.

In this study, data with more than 25% missing values were eliminated, and the missing values of the remaining data were filled according to the type of variable. The mean or median was used for numerical variables, and the mode was used for categorical variables. Categorical variables are expressed as frequency (percentage), while continuous variables are presented as mean ± standard deviation or median (interquartile range). The Mann-Whitney U test was used for continuous variables that did not follow a normal distribution, while independent samples t-tests were applied to normally distributed variables. Chi-square tests or Fisher’s exact probability method were utilized for comparing categorical variables. Independent variables with statistical significance (*p* < 0.05) from univariate analysis were included in the binary logistic regression analysis, employing a forward stepwise regression approach to identify the variables ultimately incorporated into the prediction model. According to the partial regression coefficient corresponding to each variable, the equation is constructed, and the “rms” package of R software is used to build and publish the fall nomogram model of elderly MHD patients, while the “Dyn Nom” package and “shiny” package are used to build and publish the dynamic nomogram of elderly MHD patients’ falls, and upload and link related websites to convert it into a web calculator convenient for clinical use. The risk prediction model was verified internally by repeated sampling 1000 times using Bootstrap method. The predictive performance of internal and external validation of the model was evaluated by area under ROC curve, Hosmer-Lemeshow test(H-L Test), calibration chart and clinical decision curve analysis (DCA). *p* < 0.05 indicated that the difference was statistically significant.

## 3. Results

### 3.1. General clinical characteristics of elderly MHD patients

**Figure 1.**
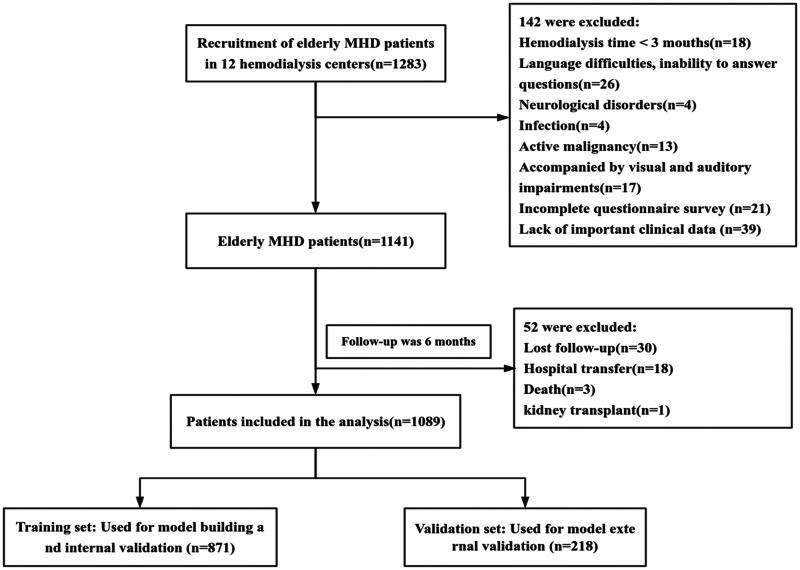
Flowchart recruitment process of the study population.

All patients were followed for 6 months from enrollment, and 52 dropped out of the study (loss of follow-up [*n* = 30], transfer to hospital [*n* = 18], death [*n* = 3], and kidney transplant [*n* = 1]). Finally, 871 patients were effectively followed up in the modeling group, including 505 males (58.0%) and 366 females (42.0%). The age was 69.924 ± 7.832 years, and the incidence of MHD falls was 31.11% (271/871) in the modeling group. A total of 218 patients were effectively followed up in the verification group, including 124 males (56.9%) and 94 females (43.1%). The age was 69.036 ± 7.403 years, and the incidence of MHD falls in the verification group was 35.32% (77/218). Comparisons of general characteristics between the modeling and validation groups revealed no statistically significant differences (*p* > 0.05). The patient recruitment process is illustrated in Figure 1.

### 3.2. Univariate analysis of fall incidence in elderly MHD patients

The research participants were categorized based on the presence or absence of falls for univariate analysis. The results showed that age, sex, visual impairment, hearing impairment, sedation, hypnosis drugs, drinking history, intradialytic hypotension, frailty, cognitive impairment, depression, malnutrition, the self-care ability of daily life, sleep disturbance, and serum albuminhad strong significant relationship (*p* < 0.05) with the occurrence of falls. The general characteristics of the dataset are detailed in Table 1.

**Table 1.** Baseline characteristics and univariate analysis of the study population.

Variables	Total (*n* = 871)	Non-­faller (*n* = 600)	Faller (*n* = 271)	Statistic	*P* Value
Sex				11.757	.001
Male	505(58%)	371(61.8%)	134(49.4%)		
Female	366(42%)	229(38.2%)	137(50.6%)		
Age (years)	69(63.00,75.0)	68(61.00,73.00)	74(68.00,80.00)	−8.974	<.001
Duration of dialysis (month)	34(12,60)	34(12.00,60.00)	31(12.00,57.00)	−0.494	.621
BMI (kg/m^2^)	22.4(20.10,24.60)	22.5(20.20,24.68)	22(19.80,24.50)	−1.223	.222
Number of chronic diseases	2.00(1.00,2.00)	2(1,2)	2(1,2)	−0.307	.759
Educational level				2.872	.412
Primary and below	308(35.3%)	202(33.7%)	106(39.1%)		
junior high school	321(36.9%)	230(38.3%)	91(33.6%)		
Senior high school	135(15.5%)	95(15.8%)	40(14.8%)		
Specialist and above	107(12.3%)	73(12.2%)	34(12.5%)		
Marital status				4.872	.181
Married	744(85.4%)	518(86.3%)	226(83.4%)		
Unmarried	5(0.6%)	5(0.8%)	0		
Divorced	20(2.3%)	14(2.3%)	6(2.2%)		
Widowed	102(11.7%)	63(10.5%)	39(14.4%)		
Living situation				2.108	.302
Live with family	807(92.7%)	552(92.0%)	255(94.1%)		
Living alone	62(7.1%)	47(7.8%)	15(5.5%)		
Residential care facility	2(0.2%)	1(0.2%)	1(0.4%)		
Monthly household income				5.357	.147
<1000	92(10.6%)	73(12.2%)	19(7.0%)		
1000–3000	303(34.8%)	206(34.3%)	97(35.8%)		
3001–5000	299(34.3%)	203(33.8%)	96(35.4%)		
>5000	177(20.3%)	118(19.7%)	59(21.8)		
Current smoking				3.797	.050
Yes	105(12.1%)	81(13.5%)	24(8.9%)		
No	766(87.9%)	519(86.5%)	247(91.1%)		
Current alcohol consumption				0.016	.898
Yes	43(4.9%)	30(5.0%)	13(4.8%)		
No	828(95.1%)	570(95%)	258(95.2%)		
Visual impairment				34.865	<.001
Yes	370(42.5%)	215(35.8%)	155(57.2%)		
No	501(57.5%)	385(64.2%)	116(42.8%)		
Hearing impairment				26.386	<.001
Yes	209(24.0%)	114(19%)	95(35.1%)		
No	662(76%)	486(81%)	176(64.9%)		
physical exercise				31.485	<.001
Never	364(41.8%)	214(35.7%)	150(55.4%)		
Occasionally	231(26.5%)	169(28.2%)	62(22.9%)		
Frequently	276(36.7%)	217(36.2%)	59(21.8%)		
Intradialytic hypotension				75.797	<.001
Yes	230(26.4%)	106(17.7%)	124(45.8%)		
No	641(73.6%)	494(82.3%)	147(54.2%)		
Sedative-hypnotic drug				4.971	.026
Yes	227(26.1%)	143(23.8%)	84(31%)		
No	644(73.9%)	457(76.2%)	187(69%)		
Frailty				68.811	<.001
Yes	331(38%)	173(28.8%)	158(58.3%)		
No	540(62%)	427(71.2%)	113(41.7%)		
Cognitive impairment				95.097	<.001
Yes	263(30.2%)	120(20%)	143(52.8%)		
No	608(69.8%)	480(80%)	128(47.2%)		
Depression					
Yes	333(38.2%)	157(26.2%)	176(64.9.%)	118.873	<.001
No	538(61.8%)	443(73.8%)	95(35.1%)		
Malnutrition				60.662	<.001
Normal	10(1.1%)	8(1.3%)	2(0.7%)		
Mild	589(67.7%)	452(75.3%)	137(50.6%)		
Moderate	252(28.9%)	134(22.3%)	118(43.5%)		
Severe	20(2.3%)	6(1%)	14(5.2%)		
Sleep quality				32.407	<.001
Very good	113(13.0%)	93(15.5%)	20(7.4%)		
Better	332(38.1%)	247(41.2%)	85(31.4%)		
Average	309(35.5%)	199(33.2%)	110(40.6%)		
Poor	117(13.4%)	61(10.2%)	56(20.7%)		
Activities of daily living	100.00(90.00,100.00)	100(90.00,100.00)	90(75.00,100.00)	−8.07	<.001
Social support	26.00(22.00,30.00)	26(22.00,30.75)	25(21.00,29.00)	−2.095	.036
Hemoglobin (g/L)	110.00(98.00,121.00)	110.00(97.00,121.00)	111.00(99.00,121.00)	−0.568	.570
Hypersensitive c-reactive protein(mg/L)	6.91(2.60,14.46)	7.62(2.60,14.56)	5.60(2.50,14.46)	−0.434	.664
Serum Albumin(g/L)	39.40(36.40,41.90)	39.75(36.93,42.10)	38.90(35.50,41.30)	−3.245	<.001
Serum Phosphorus(mmol/L)	1.63(1.27,2.00)	1.65(1.31,2.02)	1.55(1.31,2.02)	−1.897	.058
Serum Calcium(mmol/L)	2.17(2.05,2.3)	2.18(2.04,2.32)	2.16(2.06,2.28)	−1.015	.310
Serum Sodium(mmol/L)	139.00(136.80,141.10)	139.00(136.90,141.20)	138.80(136.40,141.00)	−1.242	.214
Total Cholesterol(mmol/L)	3.70(2.98,4.28)	3.72(3.05,4.29)	3.60(2.93,4.28)	−0.986	.324
parathyroid hormone(pg/ml)	246.81(132.20,405.50)	257.90(132.98,410.16)	232.60(128.80,389.70)	−1.623	.105
Triglycerides(mmol/L)	1.61(1.06,2.50)	1.66(1.08,2.62)	1.50(1.02,2.31)	−1.784	.074
Blood Urea Nitrogen(mmol/L)	19.88(14.40,23.50)	19.96(14.41,24.31)	19.88(14.12,21.96)	−1.852	.064
Serum Creatinine(umol/L)	702.40(466.60,916.00)	721.75(506.28,946.26)	644(385.00,846.60)	−4.245	<.001

### 3.3. Multivariate analysis of fall incidence in elderly MHD patients

Using the presence of fall in elderly MHD patients as the dependent variable, multifactorial binary logistic regression analysis was conducted with statistically significant variables from univariate analysis as independent variables. The results showed that age, gender, impaired vision, intradialytic hypotension, cognitive impairment, and depression were independent influences on the occurrence of falls in elderly MHD patients (*p* < 0.05), as shown in Table 2. The logistic risk prediction model was constructed based on the β coefficients and constants of the respective variables as follows:

$$P=\frac{1}{1+\;\text{exp}\;\left[-\left(\begin{array}{l} -\;6.07-0.421\cdot \text{Gender}(\text{male})\\ +\;0.410\cdot \text{Visual Impairment}(\text{yes})\\ +\;0.051\cdot \text{Age}\;+\;1.197\cdot \text{Intradialytic Hypotension}\\ +\;1.511\cdot \text{Deperssion}(\text{yes})\\ +\;1.14\cdot \text{Cognitive Impairment}(\text{yes}) \end{array}\right)\right]}$$

**Table 2.** Logistic multivariate regression analysis of falls in patients.

Variables	β	SE	Wald χ^2^	OR	95%CI	*p* Value
Sex(Male)	−0.421	0.18	5.495	0.656	0.461-0.933	.019
Visual impairment(Yes)	0.410	0.182	5.063	1.507	1.054-2.154	.024
Age	0.051	0.012	16.905	1.053	1.027-1.079	<.001
Intradialytic hypotension(Yes)	1.197	0.194	37.966	3.311	2.262-4.845	<.001
Depression(Yes)	1.511	0.181	69.838	4.533	3.18-6.461	<.001
Cognitive impairment(yes)	1.14	0.191	35.604	3.126	2.15-4.546	<.001
Constant	−6.07	0.885	47.074	——	——	.002

### 3.4. Construction of fall risk prediction model in elderly MHD patients

**Figure 2.**
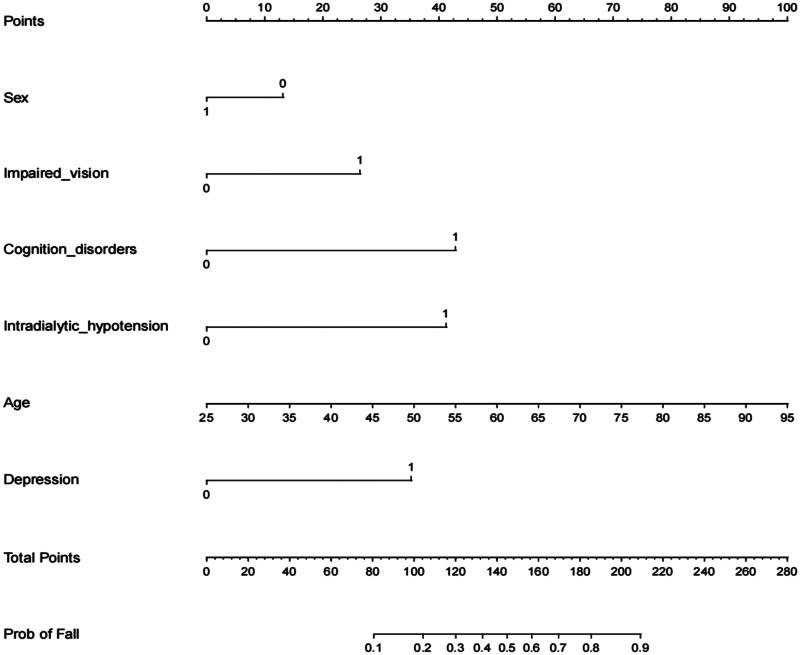
Nomogram for predicting the risk of fall in elderly MHD patients.

**Figure 3.**
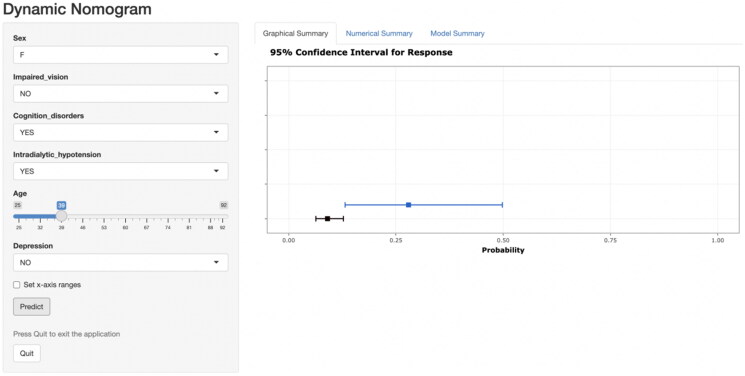
Application of a web-based calculator based on a dynamic fall nomogram in elderly MHD patients.

Based on Logistic regression analysis results, a prediction model was established and a visual nomogram was drawn, as shown in Figure 2. For each selected variable, different values correspond to specific scores (0–100 points) on the score scale at the top of the nomogram, determined by drawing a vertical line. The scores of all variables are then summed to obtain a total score, which is used to determine the risk probability of falls in elderly MHD patients. To further simplify the calculation process, the “Dyn Nom” package of R4.3.1 software was used to create an online calculator that predicts the risk probability of falls in these patients. The calculator can be accessed at the following URL: https://yixueliexiantufall.shinyapps.io/DynNomapp/, with an example shown in Figure 3.

### 3.5. Validation of the fall risk prediction model for elderly MHD patients

#### 3.5.1. Internal validation of the modeling group

**Figure 4.**
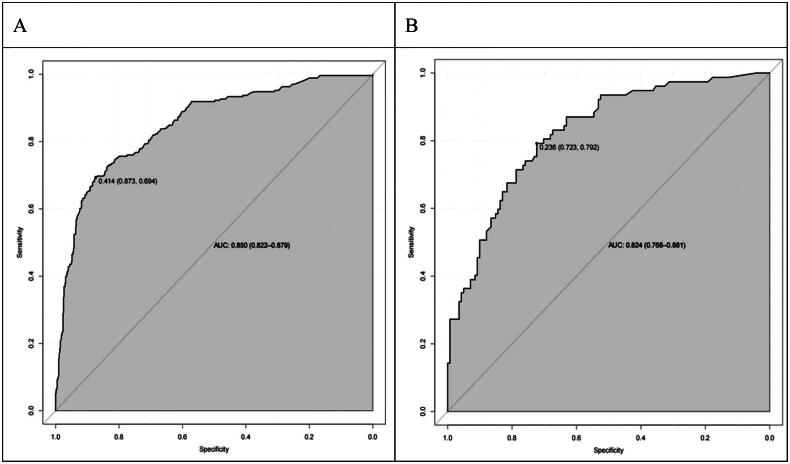
Internal validation of the depression prediction model for elderly MHD patients: (A) Internal validation ROC curve; (B)External validation ROC curve.

**Figure 5.**
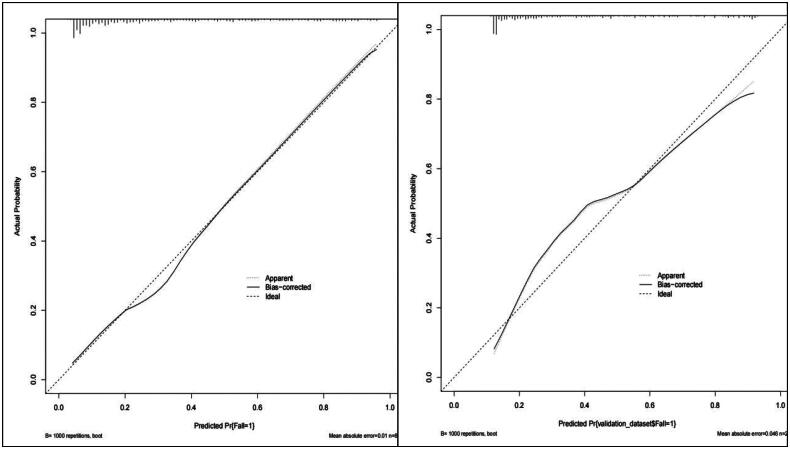
Calibration curve of fall prediction model in elderly MHD patients: (A) Internal validation calibration curve; (B) External validation calibration curve.

The Bootstrap method was used to perform 1,000 repeated samplings to evaluate the model’s discrimination and calibration. The AUC was 0.850, with a 95% CI of (0.822, 0.879), as shown in Figure 4(A). The model’s sensitivity was 0.579, and its specificity was 0.900. The detailed performance of the model is presented in Table 3. The calibration curve indicated that the calibration curve closely matched the ideal curve, with an average absolute error of 0.01, as shown in Figure 5(A). The Hosmer-Lemeshow test yielded (X^2^=2.685, *p* = 0.953), (*p* > 0.05).

**Table 3.** Predictive performance of the model.

AUC (95% CI)	Sensitivity	Specificity	Accuracy	F1-score
0.850(0.822,0.879)	0.579	0.900	0.800	0.641

#### 3.5.2. External validation

**Figure 6.**
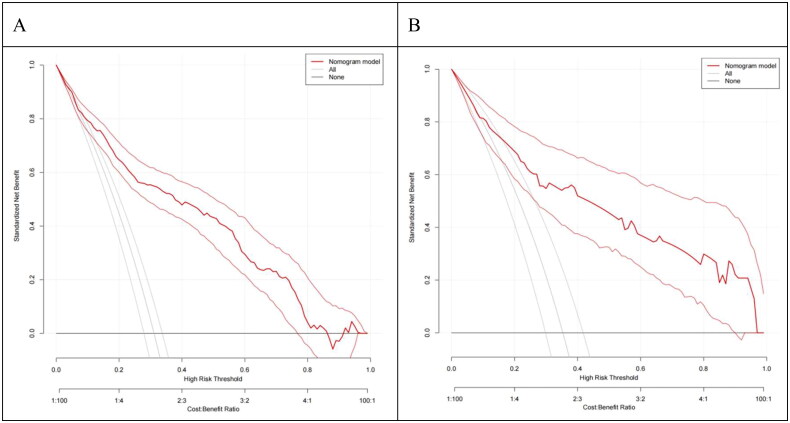
Clinical decision curve analysis of the depression prediction model for elderly MHD patients: (A) Internal validation DCA curve; (B) External validation DCA curve.

External validation was conducted using 218 patients from the validation group. The results showed an AUC of 0.824, with a 95% CI of (0.768, 0.881), as shown in Figure 4(B). The calibration curve indicated a slightly lower degree of overlap with the standard curve, but the overall trend was generally consistent, with an average absolute error of 0.046, as shown in Figure 5(B). The Hosmer-Lemeshow test yielded (X^2^=1.57, *p* = 0.814), (*p* > 0.05). Additionally, the clinical decision curves demonstrated that the red curves for both the modeling group and the validation group were located in the upper right of the None line and All line, indicating that the nomogram prediction model had a higher net benefit rate and significant clinical utility, as shown in Figures 6(A) and 6(B).

## 4. Discussion

### 4.1. Incidence of falls in elderly MHD patients

Maintenance hemodialysis is one of the most widely used renal replacement therapies for patients with end-stage renal disease (ESRD). Due to prolonged hemodialysis, patients with MHD are often accompanied by complications such as weakness, malnutrition, and loss of muscle mass, and the interplay of various factors significantly increases the risk of falls in patients. In this study, we conducted an in-depth analysis of falls in elderly MHD patients, and the results showed that the incidence of falls was 31.11% in the modeling group and 35.32% in the validation group, with the overall sample reaching a 31.96% incidence of falls. This data is in general agreement with the findings of Kutner NG [22] (28.35%), indicating that elderly MHD patients are at a higher risk of falls. It is worth noting that despite the consistency of the results of this study with some of the literature, some variability exists as well. For example, the study by Hong Chan Zhang [23] reported a lower fall rate (19.64%), while van Loon IN [24] derived a higher fall rate (56.16%) through prospective analysis. These differences may be attributed to a combination of several complex factors, including, but not limited to, the length of follow-up, specific characteristics of the study population (age, gender, comorbidities), cultural and environmental differences in the study geographic area, and the size of the sample size. Differences in duration of follow-up may significantly affect the capture of fall events, whereas differences in study populations and regions may reflect the diversity of risk factors for falls among patients in different contexts.

Given the prevalence and severity of falls in elderly MHD patients, this study emphasizes the importance of fall risk assessment in this population. Healthcare professionals should be fully aware of the adverse effects of falls on patients’ quality of survival and long-term health, and accordingly develop individualized intervention strategies to effectively reduce the incidence of falls. Strategies may include enhancing safety education for patients and their families, optimizing dialysis treatment management, improving patients’ nutritional status and physical fitness, and optimizing the living environment to reduce the risk of falls.

In summary, although existing studies differ in the specific values of the incidence of falls in elderly MHD patients, they all unanimously point out the urgency and importance of the problem. Future studies should further explore the potential mechanisms and influencing factors of falls in depth, to provide a scientific basis for the development of more precise and effective prevention and treatment strategies. At the same time, fall prevention for elderly MHD patients should be strengthened in clinical practice to reduce the incidence of falls and related hazards, to improve the survival rate and quality of life of patients.

### 4.2. Analysis of risk factors for falls in elderly MHD patients

#### 4.2.1. Age and sex

In this study, age and gender were found to be independent risk factors for the occurrence of falls in elderly MHD patients, which is consistent with the findings of Kono K [25] and Wenjun Hu [26]. To analyze the reasons, with aging, elderly MHD patients generally face senile degeneration of multiple organ systems, which significantly affects their mobility, balance function, muscle mass and strength, as well as autonomy in daily activities, thus constituting an important physiological basis for the increased risk of falls. The reduced sensory acuity and prolonged reaction time associated with aging further weaken the patients’ ability to adapt and avoid unexpected situations, thus exacerbating the susceptibility to falls. In addition, gender differences were evident in this study, with elderly women with MHD showing a higher risk of falls compared with men of the same age. This finding can be attributed to female-specific physiological changes, including a significant decline in estrogen levels, which accelerates the rate of bone loss and promotes the development of osteoporosis and muscle atrophy. With the decrease in bone density and weakened muscle strength, the risk of falls in elderly women with MHD significantly increases [27]. Therefore, in clinical practice, healthcare providers should focus on elderly MHD patients by enhancing health education, implementing continuous care plans, and encouraging appropriate physical exercise to improve physical function and fall prevention abilities. For female MHD patients, the importance of bone health management should be emphasized, including guidance on appropriate calcium supplementation and early screening and treatment of osteoporosis to slow bone loss and reduce fall risk.

#### 4.2.2. Visual impairment

The results of this study showed that visual impairment had a significant effect on the occurrence of falls in elderly MHD patients, with an OR of 1.507, indicating that elderly MHD patients with visual impairment tend to be more prone to falls compared to normal elderly MHD patients, which is basically in line with the results of the studies conducted by Wang HH [28] and Liv Guilin [29]. With age, the human visual system inevitably undergoes degenerative changes, resulting in the problem of visual impairment becoming more prevalent in the elderly population. Coupled with the fact that in recent years, diabetic nephropathy, as one of the important causes of chronic kidney disease, and its complication diabetic retinopathy have significantly exacerbated the visual impairment status of elderly MHD patients [30]. Existing studies have confirmed [31] that impairment of visual function can directly affect gait stability in older adults, as evidenced by unstable gait patterns and decreased balance. For elderly MHD patients, the loss of visual adjustment and visual information processing ability further weakens their ability to maintain postural balance in complex environments, thus increasing the risk of falls. Thus, for this high-risk group, medical personnel should strengthen the assessment and monitoring of patients’ visual function, and timely detect and diagnose visual impairment problems. Secondly, through visual training and ophthalmologic treatment, efforts should be made to enhance the visual function level of patients and improve their gait stability. At the same time, patient education should be strengthened to raise their awareness of the importance of vision protection and fall prevention, and to enhance treatment compliance, to jointly promote the improvement of patients’ quality of life and the reduction of fall risk.

#### 4.2.3. Intradialytic hypotension

The study results showed that intradialytic hypotension was an important risk factor for falls in elderly MHD patients (OR = 1.507), which was consistent with the results of Zhao Li [32]. Intradialytic hypotension is one of the most common and serious acute complications in MHD patients, with its high incidence (6.7% to 39.9%) [33,34] and close association with multiple adverse clinical outcomes [35], which has attracted wide attention. During intradialytic hypotension, patients often appear sweating, dizziness, blurred vision, constipation or incontinence and other symptoms, significantly decreased blood pressure can also appear angina pectoris, arrhythmia, vomiting, lethargy and even muscle cramps, dyspnea and transient syncope and other serious reactions. These symptoms not only greatly affect the quality of life of patients, but also directly increase the risk of falls in elderly MHD patients, especially in the process of dialysis or just after dialysis, patients are more vulnerable to accidental falls. Therefore, to reduce the incidence of intradialytic hypotension and falls in elderly MHD patients, medical personnel should strengthen the dietary guidance for patients, through reasonable control of dietary intake, reduce unnecessary weight gain during dialysis, thereby reducing hemodynamic fluctuations during dialysis and reducing the risk of hypotension. Secondly, according to the specific situation of the patient, develop a personalized antihypertensive drug treatment plan, and closely monitor the patient’s response during the treatment process, and timely adjust the drug dose or type. In addition, it is equally important to optimize the dialysis program, including adjusting the composition of dialysate, dialysis time, dialysis frequency and other parameters to better adapt to the physiological needs of patients and reduce the occurrence of dialysis-related complications.

#### 4.2.4. Cognitive impairment

The study results indicate that cognitive impairment is an independent risk factor for predicting falls in elderly MHD patients, with cognitively impaired patients having a 3.126 times higher risk of falling than those without impairment, consistent with the findings of Li [36]. Cognitive impairment, characterized by mild memory loss, concentration difficulties, and reduced decision-making ability, is one of the most common issues in an aging society [37]. The incidence of cognitive impairment in elderly hemodialysis patients ranges from 30% to 60% [38]. Cognitive impairment can have long-term effects on elderly MHD patients’ health, self-management, and daily life. Studies have shown [39] that the decline in executive function, attention, reaction speed, and information processing ability due to cognitive impairment is a major reason for balance function impairment, disrupted walking rhythm, slowed walking speed, and increased gait variability in elderly patients, collectively contributing to an increased risk of falls. Furthermore, abnormalities in central nervous system function may lead to muscle weakness, further triggering falls [40]. Given the high prevalence of cognitive impairment and its impact on fall risk in elderly MHD patients, strengthening the assessment and screening of cognitive function and implementing targeted interventions are crucial for improving patients’ quality of life and reducing fall risk. It is important to note that patients in the early stages of cognitive impairment retain a degree of cognitive plasticity, providing an opportunity to improve cognitive function through interventions. Healthcare providers should use a combination of cognitive training, physical exercise, dietary adjustments, and emotional management interventions to enhance patients’ cognitive and physical functions, effectively reducing the occurrence of falls.

#### 4.2.5. Depression

This study shows that depression is a risk factor for falls in elderly MHD patients (OR = 2.475), indicating that patients with depressive symptoms have a higher incidence of falls. Previous studies have also pointed out [41] that depression in elderly MHD patients is closely related to falls. Maintenance hemodialysis, as the primary treatment for end-stage renal disease, effectively prolongs survival, but its frequent treatment requirements, complex complication management, and profound impact on daily life place a heavy psychological and economic burden on patients. These factors contribute to the prevalence of depression in elderly MHD patients, with a prevalence rate ranging from 15% to 52% [42], far exceeding that of the general population. Depression is not only characterized by low mood but also by cognitive decline, attention deficits, and reduced executive function, which directly affect daily life activities, particularly walking, balance, and coordination, thereby increasing fall risk [43,44]. Additionally, research has shown [45] that depression can lead to reduced physical activity and muscle weakness, further exacerbating gait instability and balance impairment, triggering falls. Therefore, healthcare providers need to strengthen early screening and assessment of depressive symptoms for timely intervention. Regularly providing psychological support services, such as counseling and emotional management, can help alleviate psychological stress. Organizing diverse recreational activities and medical knowledge lectures, encouraging social interaction, and improving quality of life and self-efficacy can holistically maintain patients’ physical and mental health and reduce the occurrence of falls and other adverse events.

### 4.3. Prediction model of falls

Based on the results of multivariate Logistic regression analysis, this study established a nomogram of the fall prediction model for elderly MHD patients. Six variables including age, gender, visual impairment, intradialytic hypotension, cognitive impairment and depression were finally included in the model. In order to evaluate the predictive performance of the model, the differentiation, calibration and clinical validity of the model were evaluated through spatial validation and time period validation in internal and external validation. Specifically, the ROC curve was used to evaluate the prediction effect of the model. When the AUC was 0.5 ∼ 0.7, the prediction ability of the model was low. When the AUC is 0.7 ∼ 0.9, the prediction ability of the model is better. When AUC >0.9, it indicates that the model has strong predictive ability. The AUC of internal validation and external validation of the model in this study are 0.850, 95%CI (0.822,0.879) and 0.824, 95%CI (0.768,0.881), respectively. when the optimal risk cutoff value is 0.414, the model’s specificity is 0.579, and the sensitivity is 0.900, which suggests that the predictive efficacy of the model is good. In addition, the calibration curve analysis results of the model showed that the calibration curve was highly coexisting with the ideal curve regardless of internal verification or external verification, and the H-L test results showed *p* > 0.05, which further indicated that the model had high diagnostic value and good fitting degree. The DCA analysis showed that the net benefit of the intervention using this model was greatest when the predicted risk was in a specific range of 16% to 85%, that is, preventive intervention for patients could bring significant clinical benefits. The fall risk prediction model of elderly MHD patients constructed in this study, with its scientific construction method, good prediction performance, and significant clinical value, provides a powerful tool for individualized and accurate fall risk assessment, which is helpful to guide clinical practice, optimize patient management strategies, and reduce the incidence of fall events.

### 4.4. Implications for MHD management

As an intuitive and visual risk prediction graph, Nomogram has been widely used in medical research and clinical practice in recent years, so that medical personnel can directly use this graph to calculate the values of each variable, to obtain the predicted total score and corresponding risk probability. In this study, the fall prediction model for elderly MHD patients was visualized in the form of a nomogram, and the complex regression equation was transformed into a simple and visual graph, so that the operator could intuitively understand the impact of various factors on the prediction results and their interactions, which significantly increased the readability of the outcome of the prediction model. In addition, to further promote the popularization and application of the nomogram model in clinical practice, this study carried out technical deepening and developed a convenient web-based calculator. The calculator is easy to use and allows you to instantly generate a personalized fall risk probability report by entering the values of a patient’s six key predictors. This innovation not only simplifies the prediction process and improves work efficiency, but also ensures the accuracy and consistency of the prediction results, providing a strong technical support for the fall risk assessment and management of elderly MHD patients.

## 5. Limitations

Although this study has made positive progress in building a fall risk prediction model for elderly MHD patients, its limitations should be addressed. First, the research samples are mainly concentrated in the same province, and the regional representation is relatively limited, which may affect the applicability of the model in a wider population. Future research should focus on multi-center and cross-regional sample collection to enhance the universality and generalization value of the model. Second, due to time and manpower constraints, the follow-up period of this study was only half a year, which may not fully reveal the impact of certain long-term factors on fall risk. Therefore, extending the follow-up period and exploring the long-term relationship between each factor and the fall outcome will be an important direction to optimize the prediction efficiency of the model. Finally, some potential predictors were not included in this study due to regional differences or patient compliance, limiting the comprehensiveness of the model. Future studies should consider including more objective factors, and further improve the accuracy and practicability of the fall risk prediction model by expanding the scope of variables and optimizing the model structure.

## 6. Conclusion

The results of this study showed that age, gender, visual impairment, hypotension in dialysis, cognitive impairment, and depression are independent risk factors affecting the occurrence of falls in elderly maintenance hemodialysis patients, and based on these risk factors, we constructed a fall nomogram prediction model and developed a web page calculator, which was internally and externally validated, and comprehensively demonstrated that the model is of good scientific validity and utility. It can be used as a specific prediction tool for falls in elderly MHD patients and provide guidance for early clinical identification of high-risk patients.

## Data Availability

The datasets generated during and analyzed during the current study are not publicly available but are available from the corresponding author on reasonable request.
